# A Collaborative Multiple Stressor Approach for Identifying Spatial Heterogeneities in Wildlife Health and Conservation Priorities

**DOI:** 10.1093/icb/icaf123

**Published:** 2025-06-28

**Authors:** Molly C Simonis, Sarah Ciarrachi, Kristin E Dyer, Meagan Allira, Bret Demory, Jaleel Zubayr, Dakota Van Parys, Kimberlee Whitmore, Katie Fitzgerald, Kevin T Castle, Tanya A Dewey, Joy M O'Keefe, Riley F Bernard, Matthew M Chumchal, Catherine G Haase, Jeffrey T Foster, Daniel J Becker

**Affiliations:** School of Biological Sciences, University of Oklahoma, Norman, OK 73019, USA; College of Forestry, Wildlife and Environment, College of Veterinary Medicine Department of Pathobiology, Auburn University, Auburn, AL 36849, USA; Department of Biological Sciences, Pathogen and Microbiome Institute, Northern Arizona University, Flagstaff, AZ 86011, USA; School of Biological Sciences, University of Oklahoma, Norman, OK 73019, USA; School of Biological Sciences, University of Oklahoma, Norman, OK 73019, USA; School of Biological Sciences, University of Oklahoma, Norman, OK 73019, USA; School of Biological Sciences, University of Oklahoma, Norman, OK 73019, USA; Department of Biology, Austin Peay State University, Clarksville, TN 37044, USA; Center for Excellence in Field Biology, Austin Peay State University, Clarksville, TN 37044, USA; Department of Biology, Texas Christian University, Fort Worth, TX 76109, USA; Department of Natural Resources and Environmental Sciences, University of Illinois Urbana-Champaign, Urbana, IL 61801, USA; Wildlife Veterinary Consulting, Livermore, CO 80536, USA; Department of Biology, Colorado State University, Fort Collins, CO 80523, USA; Department of Natural Resources and Environmental Sciences, University of Illinois Urbana-Champaign, Urbana, IL 61801, USA; Department of Zoology & Physiology, University of Wyoming, Laramie, WY 82071, USA; Department of Biology, Texas Christian University, Fort Worth, TX 76109, USA; Department of Biology, Austin Peay State University, Clarksville, TN 37044, USA; Center for Excellence in Field Biology, Austin Peay State University, Clarksville, TN 37044, USA; Department of Biological Sciences, Pathogen and Microbiome Institute, Northern Arizona University, Flagstaff, AZ 86011, USA; School of Biological Sciences, University of Oklahoma, Norman, OK 73019, USA

## Abstract

Wildlife face a number of extrinsic stressors, such as habitat loss, pathogen infections, and contaminant exposure, which can increase the energy needed to maintain optimal health and survival. These multiple extrinsic stressors can also occur simultaneously during intrinsically stressful life stages such as reproduction, migration, or hibernation. To fully understand how to support healthy wildlife populations, we must quantify physiological and immunological phenotypes across a variety of stressors. We pose a framework for conducting field studies to collect individual-level samples that can be used for measuring physiological and immunological phenotypes as well as the potentially stressful intrinsic and extrinsic drivers of those phenotypes. We suggest that collaborative efforts should then be made to create broader, spatially coordinated hypotheses for determining patterns of wildlife health under intrinsically stressful time periods and across extrinsically stressful landscapes. We provide an example and preliminary findings for this multi-stressor, collaborative, and spatially coordinated approach with an ongoing study of North American bat health. Quantifying direct and critical measures of wildlife health and identifying key intrinsic and extrinsic stressors that drive physiological and immunological phenotypes will provide broad targets for conservation strategies and where and when those strategies should be prioritized in the future.

Wildlife health encompasses the ability of wild animals to adapt to environmental change through physiological and immunological responses to abiotic and biotic stressors (Deem et al. 2001; Acevedo-Whitehouse and Duffus 2009; Stephen 2014). Quantifying wildlife health can provide a direct view for how wildlife respond and adjust to energetically costly changes in their environments, such as emerging infectious diseases and human-induced land use change (Deem et al. 2001; Acevedo-Whitehouse and Duffus 2009; Stephen 2014). Physiological and immunological phenotypes underlie and can predict pathogen susceptibility, survival and fecundity, and long-term population viability, all of which are crucial to wildlife conservation (Acevedo-Whitehouse and Duffus 2009). Multiple intrinsic and extrinsic stressors can threaten wildlife health and survival (Munns 2006; Thiel et al. 2008; O'Connor et al. 2009; Allen et al. 2011; Eidels et al. 2016; Hill et al. 2016; McGuire et al. 2017). For example, North American bats, such as *Myotis lucifugus*, infected with *Pseudogymnoascus destructans* have increased torpid metabolic rates and arousal frequencies, which can cause them to starve during physiologically challenging periods, such as winter hibernation (Reeder et al. 2012; McGuire et al. 2017). How wildlife respond to intrinsic and extrinsic stress can additionally vary by individuals, species, and the multiple landscapes they inhabit (Gervasi et al. 2015; Schmitt et al. 2017; Becker et al. 2020, 2023). To mitigate the impact of multiple intrinsic or extrinsic stressors on threatened species, conservation efforts extend species protections to the land to ensure quality habitats for species survival (United States 1983). However, population monitoring within protected lands does not require direct measures of individual wildlife health, nor does it account for the heterogeneity of surrounding landscapes and habitats wildlife encounter (Fahrig et al. 2011). We cannot fully understand how to support healthy wildlife populations without directly measuring physiology and immunology across varying spatial features and environments. To overcome these challenges, coordinating field studies that quantify these phenotypes as critical measures of wildlife health can identify the relative importance of many intrinsic and extrinsic stressors and, thus, identify critical targets for conservation strategies.

Wildlife endure multiple intrinsic and extrinsic stressors throughout their lifetime that can alter their physiology and immunity. Wildlife experience natural, intrinsic periods of physiological stress and increased energy expenditures, especially during reproduction and migration (Wikelski et al. 2003; Richardson et al. 2018). However, extrinsic stressors such as resource scarcity, harsh abiotic conditions (e.g., winter or drought), habitat loss, contaminant exposure, and pathogens can inflate energetic demands (Thiel et al. 2008; O'Connor et al. 2009; Allen et al. 2011; Eidels et al. 2016; Hill et al. 2016; McGuire et al. 2017; Holden et al. 2022; Vaziri et al. 2024). Additionally, extrinsic stressors can have compounding effects on wildlife by modifying the physiological and immunological phenotypes that help combat their effects and minimize infection risks across diverse landscapes. For example, sewage water contamination decreases circulating neutrophils and increases lymphocytes in desert bats (*Pipistrellus kuhlii*) within a month of drinking contaminated water daily (Pilosof et al. 2014). For amphibians, poor water quality may increase the presence of *Batrachochytrium dendrobatidis* and thus increase the risk of chytridiomycosis (Jacinto-Maldonado et al. 2023). Further, infection-induced mortality could reduce wildlife population size when pathogen transmission in polluted landscapes is equal to or greater than in natural habitats (Sánchez et al. 2020). While effects vary by individuals, species, and context, the compounding effects of intrinsic and extrinsic stressors could be detrimental to wildlife populations. Here, we pose a framework and a call for increased spatially coordinated monitoring in which teams pair measurements of multiple stressors with physiological and immunological biomarkers to build a holistic understanding of wildlife health.

## Measuring wildlife health

Individual-level responses to intrinsic and extrinsic stressors can be energetically costly, potentially disrupting allostasis (i.e., energy budgets; McEwen and Wingfield 2003) and thus, overall health. When considering that both intrinsic and extrinsic stressors act simultaneously on wildlife, compounding energy expenditures from multiple physiological and immunological responses (i.e., allostatic load) can threaten an individual’s survival (Beckie 2012; Seeley et al. 2022) and increase risks associated with pathogen shedding and transmission (Plowright et al. 2024). Multiple physiological and immunological biomarkers are used to measure the impact of stressors on wildlife allostatic loads (e.g., energy expenditures [basal metabolic rates, resting metabolic rates, torpid metabolic rates], baseline glucocorticoid concentrations, white blood cell counts [see example in Box 1], heat-shock proteins; Edes et al. 2018). While researchers may measure different physiological or immunological biomarkers depending on project aims, laboratory capacity, sampling logistics, and funding, incorporating direct measurements of health in relation to allostatic load is critical for linking hypothesized stressors to phenotypes.

**Box 1. We piloted a longitudinal project aimed at identifying intrinsic and extrinsic drivers of North American bat cellular immunity**. We captured female bats (*n* = 55 across five species) in Tennessee, Oklahoma, and Arizona in summer 2023 (A; Table S1). From each bat, we collected individual-level data and samples, including (but not limited to) bat holding time, body mass, sex, reproductive status (intrinsic stressor), whole blood, and fur. Using these samples, we quantified NL ratios (proxy for chronic stress) and micronuclei intensities (extrinsic stressor) from blood smears, detected *Bartonella spp*. infections from whole blood (extrinsic stressor), and quantified THg concentrations from fur (extrinsic stressor). We also determined species-specific foraging distances to calculate foraging home range areas (Table S2) and used the US Geological Survey’s 2019 National Land Cover Database (Dewitz and US Geological Survey 2021) to determine land use proportions available for foraging within those foraging home range areas (used as buffers from capture locations). Land use proportions within species-specific foraging home range areas were reduced to two PCA axes (extrinsic stressors): PC1 loaded negatively for developed lands and positively for natural, barren lands, while PC2 loaded negatively for croplands and positively for forests and shrubs (Table S3; Fig. S1). For preliminary analyses, we created 20 generalized linear models (GLMs) for NL ratios, with each model containing a main effect of bat holding time as a precision covariate and either a single interaction term of an intrinsic and extrinsic stressor or without an interaction term of those same variables (main effects only; Table S4). These models thus considered both additive and interactive effects between individual-level and spatial drivers. We compared GLMs using the Akaike information criterion corrected for small sample sizes (AICc). When exploring preliminary results from the top two models (ΔAICc ≤ 3.04), we found strong relationships between NL ratios, intrinsic or extrinsic stressors, and holding time. NL ratios differed by reproductive status (B), showed potential trends for increasing with micronuclei intensities (C), and showed potential trends for decreasing with PC1 (greater NL ratios associated with developed lands and lower NL ratios associated with natural, barren lands; D) after adjusting for holding time. While the modest dataset from our pilot season limits our ability to draw strong conclusions, and space and species identity are challenging to differentiate within pilot data, we still identified key correlates (reproduction, genotoxic effects of pollutant exposure, and spatial features) of wildlife health (NL ratios). This project expanded to four new sites in 2024 (in Illinois, Missouri, Colorado, and Wyoming), and the larger individual- and site-level sample size should enable more robust inference with less confounding spatial and taxonomic data and fewer influences of outliers. We will continue to spatially and taxonomically expand this study over time within North American bats and encourage other researchers interested in participating in this project at their spring/summer/fall field sites to contact the corresponding author (MCS).

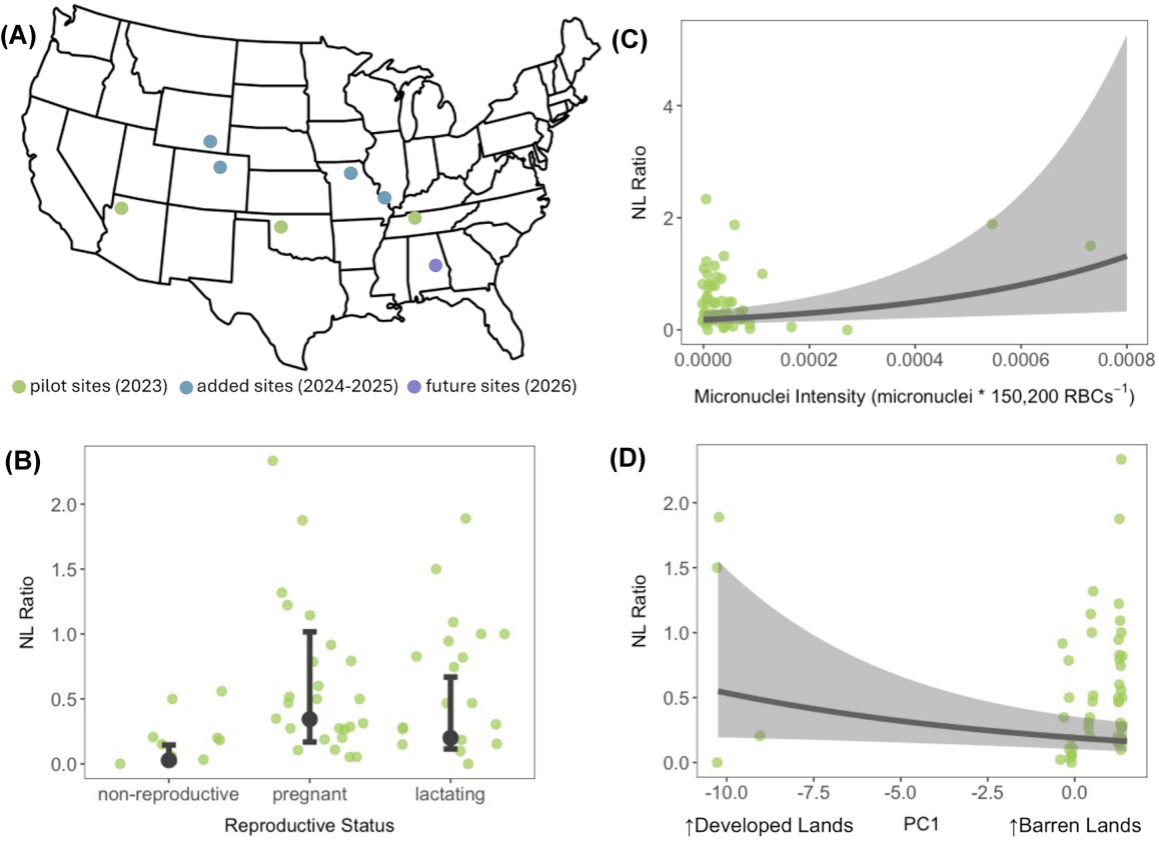

## Spatial heterogeneities of wildlife health and extrinsic stressors

Identifying the spatial drivers of physiological and immunological changes can inform an understanding of how environmental features impact wildlife health (Becker et al. 2020). For example, glucocorticoid concentrations are greater within human-disturbed landscapes for mammals globally compared to natural lands (Mirante et al. 2025). There is spatial variation in North American bat body mass, fat, and energy requirements for hibernation due to gradients of wintering temperatures (Hranac et al. 2021). Additionally, house sparrow (*Passer domesticus*) inflammatory responses vary by latitude within and beyond their native range (Martin et al. 2004; Becker et al. 2023). Regional land use features also predict changes in wildlife physiology and immunity. For example, cortisol levels in coyotes (*Canis latrans*) increase with land development across the Chicago Metropolitan Area (Robertson et al. 2023), tree swallows (*Tachycineta bicolor*) have greater bacterial killing ability in their plasma with increasing farmland intensity (Schmitt et al. 2017), and preliminary findings from our group suggest neutrophil-to-lymphocyte (NL) ratios vary with land use proportions within a species’ foraging area (Box 1).

Variations across individual-level physiological and immunological phenotypes can be determined for specific environmental factors, such as site-specific pollutants, including heavy metals, pesticides, and forever chemicals. Neutrophil counts increase with total mercury (THg) concentrations in bat fur (Becker et al. 2017); NL ratios vary with potential pesticide exposure in Neotropical bats (Sandoval-Herrera et al. 2023); micronuclei intensities (genotoxic effects of pollutant exposure) increase with environmental pollution across many taxa (Fernandez et al. 1993; Naidoo et al. 2015; Calao-Ramos et al. 2021; Sandoval-Herrera et al. 2021), including our own multi-species bat data (Box 1); microplastics alter immune gene expression in crabs and corals (Liu et al. 2019; Bove et al. 2023); and cortisol increases with PFAS pollution intensity in fish (Schumann et al. 2024). These examples of spatially correlated physiological and immunological phenotypes can also drive differences in pathogen transmission and infection across landscapes (Hawley and Altizer 2011). Additionally, the examples above provide evidence that we have clear assay targets across various wildlife species. Therefore, as wildlife physiologists and ecoimmunologists, we are in a position to better understand wildlife health ranging from population to individual levels. Combining individual-level and spatial drivers of wildlife health with appropriate measurements of each could provide greater insights for deriving broader patterns of wildlife health heterogeneity and for characterizing infection risks associated with those patterns.

## Stressors impact wildlife health simultaneously

Multiple intrinsic and extrinsic stressors may also have interactive effects on wildlife physiological and immunological phenotypes, although they are less frequently tested. Interactions between atrazine (herbicide) and environmental temperature are correlated with varying physiological (i.e., body mass and fat, glucocorticoid concentrations) and immune phenotypes (i.e., IgM concentrations), as well as reproductive outcomes in some lizards (Nie et al. 2023). Reef fish (*Pinguipes brasilianus*) at polluted sites have higher NL ratios than reference sites, and these differences are amplified during their reproductive season (Sueiro et al. 2020). Higher-level interactions among stressors as drivers to physiological and immune phenotypes are challenging to measure and manipulate in the field. However, building projects that collect comprehensive samples (i.e., multiple samples from each individual captured at a single time point) that measure both health and specific stressors of interest could highlight key interactions across intrinsic and extrinsic stressors that drive broader patterns of wildlife health. Understanding these higher-level interactions could help us to know when and where to apply conservation actions to improve wildlife health and survival.

Due to the additive and potentially interactive effects of stressors on wildlife allostatic load, it is critical we recognize the importance of how multiple, simultaneous stressors can impact wildlife health across relevant spatial scales. Many researchers have outlined the importance of understanding how multiple extrinsic stressors impact wildlife across heterogeneous landscapes (Vinebrooke et al. 2004; Munns 2006; Ban et al. 2014; Côté et al. 2016; Tekin et al. 2020; Simmons et al. 2021; Pirotta et al. 2022). Additionally, it is important to include both intrinsic and extrinsic stressors when assessing impacts to wildlife health, because the effects of intrinsic stressors may vary seasonally (for reproduction examples, see Ruoss et al. 2019 and Box 1; for a migratory example, see Voigt et al. 2010). There is a need for frameworks that holistically address the data gaps for how wildlife health is altered by many simultaneous intrinsic and extrinsic stressors.

## A framework for building multi-stressor wildlife health datasets

**Fig. 1:**
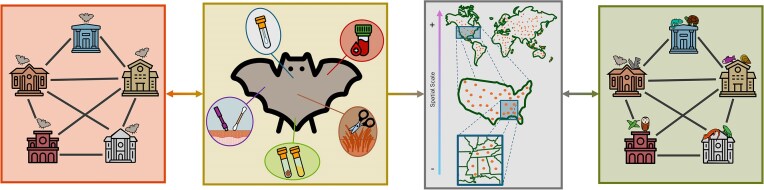
We recommend wildlife researchers communicate with each other across similar projects (far left) to collect comprehensive multi-purpose samples from each individual animal at long-term field sites (middle left). Collaborating across spatially coordinated projects would then allow for analyses at the spatial scale of choice (middle right) to summarize broad patterns of wildlife health under multiple stressors and help target conservation needs. These spatially coordinated projects within research groups could further lead to larger collaborations across phyla (far right) and feedback into identifying comprehensive phylogenetic and spatial patterns of wildlife health and conservation needs. Icons used were free downloads from the Noun Project, and created by the following artists: bat by pcymk; squirrel, owl, hummingbird, turtle, and iguana by Valerie Lamm; salamander by Yu luck; frog by Krisna Purpa; fish by Puspa Kusuma; mussel by Melissa Maury; buildings by meilia miftah choirun niswah, Denicon, and Jan Niklas Prause; global map outline by Dmitrii Lagunov; USA outline by Aidan Stonehouse; USA states outline by Joel Wisneski; fur by Saeful Muslim; blood by symbolic; sample tube by Bloger indo; swab by myiconfinder; biopsy punch Agan24; and scissors by inmyheart.

With accelerating human-induced global change, we must build robust datasets reflecting wildlife exposure to those ongoing changes. Therefore, we encourage more communication among researchers who have similar wildlife monitoring goals (Mosher et al. 2020). We recommend more comprehensive sampling in the field across projects to identify broader, spatially coordinated patterns of wildlife health under multiple stressors (Fig. 1). While comprehensive sampling may not be necessary for some individual projects, communicating with other researchers who could sample target species in a similar manner in the field can (1) cover more ground spatially and financially across both projects and (2) build collaborative capacity across interdisciplinary teams.

Within these sampling collaborations, researchers will need to determine the scales of spatial coordination that are important and cost-effective for all groups. For example, if collaborators chose to sample a single species with a broad geographic range, site specifications may be a lower priority compared to capturing across broad latitudinal or longitudinal gradients. This broad spatial approach is necessary to capture sufficient landscape variation in stressors for downstream analyses (Becker et al. 2020). For sampling widely distributed species across a broad spatial scale, capture rates may vary by region, thereby imposing additional personnel and capture effort costs for groups in areas with lower capture rates. However, if those groups are already sampling in such sites for their individual projects, contributing to these collaborative samples could maximize the personnel time spent in the field and allow benefits to outweigh costs. There are gradients of trade-offs related to sampling across different spatial scales, and collaborative groups will need to make decisions about what trade-offs benefit their shared research questions best.

Researchers may also prioritize different types of samples for landscape-scale collaborative projects depending on their research questions. Collecting comprehensive samples across projects that also serve multiple purposes can reduce the monetary costs of sampling and the number of animals that need to be sampled in the future. For example, whole blood can be used for pathogen detection and intensity, blood smears to quantify cellular immunity and micronuclei intensities, and can be spun down for plasma glucocorticoids, contaminant concentrations, and other immunological assays (e.g., immunoglobulin quantification, proteomic immune profiles; Table 1). Fur, feces, urine, and other tissue samples (e.g., a superficial biopsy punch) can be used for contaminant and glucocorticoid quantification (Table 1). Additionally, feces, urine, and skin, oral, and rectal swabs can be used for the detection of multiple different pathogens of interest (Table 1). Multi-use samples such as those given above can all be collected non-lethally, requiring small volumes or amounts of each tissue, thereby also preserving the welfare of wildlife. Collaborative sampling also increases the cost-effectiveness of fieldwork across groups (e.g., fewer travel and personnel costs to collect samples across multiple sites, lower supply costs). Scaling up collaborative sampling across broadly distributed taxa or species would provide greater insights into the drivers of wildlife health than possible through any one research group. Such collaborative datasets would allow us to broadly identify when and where wildlife conservation actions may be needed across phyla. It is crucial to consider the value of the information to be gained from comprehensive sampling in current and future wildlife health projects.

**Table 1:** Examples of tissue samples that can be collected non-lethally and their multiple uses for measuring individual-level physiological, immunological, and environmental biomarkers.

Tissue	Measure	Example reference
Whole blood	Pathogen/parasite detection	Lilley et al. (2017); Vicente-Santos et al. (2023a); Becker et al. (2025)
	White blood cell counts	Becker et al. (2021); Humphries et al. (2025)
	Micronuclei (genotoxic effects of pollutants)	Sandoval-Herrera et al. (2023)
Plasma or sera (from whole blood)	Proteome profiles	Vicente-Santos et al. (2023b); Minayo Martín et al. (2025)
	Bacterial killing ability	Field et al. (2023)
	Immunoglobulin concentrations (e.g., IgG, IgM)	Costantini et al. (2019)
	Glucocorticoid concentrations	Glucs et al. (2018); Field et al. (2023)
	Contaminant concentrations	Jessup et al. (2010)
Fur, feathers, or scales	Glucocorticoid concentrations	Glucs et al. (2018); Laberge et al. (2019)
	Contaminant concentrations	Albert et al. (2021); Simonis et al. (2025)
Saliva	Pathogen detection	Becker et al. (2022)
	Glucocorticoid concentrations	Verspeek et al. (2021); Montgomery et al. (2022)
Feces	Pathogen/parasite detection	Ferreira et al. (2021); Becker et al. (2022); Bennett et al. (2025)
	Immunoglobulin concentrations (e.g., IgG, IgM, etc.)	Ferreira et al. (2021)
	Glucocorticoid concentrations	Harper and Austad (2000); Glucs et al. (2018)
	Contaminant concentrations	Simonis et al. (2025)
Urine	Pathogen detection	Seidlova et al. (2021)
	Glucocorticoid concentrations	Verspeek et al. (2021)
	Contaminant concentrations	Shinya et al. (2022)
Skin	Pathogen detection	Bernard et al. (2015), (2017); Luciani et al. (2022)

## A working example for quantifying wildlife health under multiple stressors

To understand broad patterns for wildlife health, we piloted a longitudinal project using a broad spatial and multi-stressor approach (Box 1). We focus on North American bats, quantifying bat cellular immunity in relation to an intrinsic stressor of reproduction and extrinsic stressors of infection, contaminant exposure, and land use. In doing so, we provide proof of concept in identifying key individual-level and spatial stressors that may impact wildlife health. For our project, we chose to not focus on a particular species but instead have prioritized sampling across a broad spatial scale, targeting collaborators that were already collecting needed samples or who required training to collect such samples to benefit their individual projects. As this specific project continues over multiple years, we will expand sampling and use this approach to identify patterns for where and when bat conservation action is needed across species. This broad attempt at gaining a holistic view of wildlife health under multiple stressors can provide a roadmap for similar projects in the future and across other taxa at relevant spatial scales. Further, as others adopt the same approach, such as our example, larger collaborative efforts could bring together resources and similar comprehensive sample stores across taxa to identify global patterns for wildlife health.

## Conclusion

In conclusion, taking a broad and multi-stressor approach to quantifying wildlife health can help identify predictors of physiological and immunological phenotypes across space, thereby helping to target drivers of pathogen dynamics and conservation risks across species. We call for wildlife researchers to use similar collaborative and spatially coordinated approaches for collecting comprehensive samples from individual animals, with the goal of using those samples in multiple ways and applications (e.g., contaminant, physiology, immunology, infection) to gain more holistic views of wildlife health. We highly encourage collaborative efforts and the creation of new longitudinal projects across taxa with future goals for understanding broad patterns in wildlife health under multiple stressors.

## Supplementary Material

icaf123_Supplemental_File

## Data Availability

Due to the sensitivity of bat capture locations and ongoing data collection for the longitudinal project presented in Box 1, exact locality data are not provided. R code used for preliminary data analyses (Box 1) is available on the GitHub page of MCS (username: simonimc).

## Notes

From the symposium “Applied ecoimmunology in wildlife health and conservation” presented at the annual meeting of the Society for Integrative and Comparative Biology, January 3–7th, 2025.
