# Supplementary material for: A Collaborative Multiple Stressor Approach for Identifying Spatial Heterogeneities in Wildlife Health and Conservation Priorities
Source: Integr Comp Biol. 2025 Jun 28;65(6):1772–80. doi: 10.1093/icb/icaf123 (PMC12690475; doi:10.1093/icb/icaf123)
Supplement: icaf123_Supplemental_File [file icaf123_supplemental_file.docx]

**Supplementary Information**

**Title:** A collaborative multiple stressor approach for identifying spatial heterogeneities in wildlife health and conservation priorities

**Authors:** Molly C Simonis^1,2^, Sarah Ciarrachi^3^, Kristin E Dyer^1^, Meagan Allira^1^, Bret Demory^1^, Jaleel Zubayr^1^, Dakota Van Parys^4,5^, Kimberlee Whitmore^6^, Katie Fitzgerald^7^, Kevin T Castle^8^, Tanya A Dewey^9^, Joy M O’Keefe^7^, Riley F Bernard^10^, Matthew M Chumchal^6^, Catherine G Haase^4,5^, Jeffrey T Foster^3^, Daniel J Becker^1^

*1 School of Biological Sciences, University of Oklahoma, Norman, OK USA*

*2 College of Forestry, Wildlife and Environment and Department of Pathobiology, Auburn*

*University, Auburn, AL USA*

*3 Department of Biological Sciences, Pathogen and Microbiome Institute, Northern Arizona*

*University, Flagstaff, AZ USA*

*4 Department of Biology, Austin Peay State University, Clarksville, TN USA*

*5 Center for Excellence in Field Biology, Austin Peay State University, Clarksville, TN USA*

*6 Department of Biology, Texas Christian University, Fort Worth, TX USA
7 Department of Natural Resources and Environmental Sciences, University of Illinois*

*Urbana-Champaign, Urbana, IL USA*

*8 Wildlife Veterinary Consulting, Livermore, CO USA*

*9 Department of Biology, Colorado State University, Fort Collins, CO USA*

*10 Department of Zoology & Physiology, University of Wyoming, Casper, WY USA*

**Corresponding Author:** Molly C Simonis; molly.simonis@auburn.edu

**Journal:** *Integrative and Comparative Biology*

**Table S1**. Bat capture protocols and permits for 2023 pilot data. Bats were captured across three general sites in the US: at Fort Campbell Army Base in Tennessee, at Selman Bat Cave in Oklahoma, and in northern Arizona.

| **Site** | **State** | **Institution** | **Institutional Animal Care and Use Protocols** | **Permits** |
| --- | --- | --- | --- | --- |
| Selman Bat Cave | Oklahoma | University of Oklahoma | 2022-0198 | Oklahoma Department of Wildlife Conservation 10567389 |
| Grand Canyon | Arizona | Northern Arizona University | 21-013 | Grand Canyon National Park scientific research and collecting permit GRCA-2023-SCI-0042 and AZ_GRCA_Ciarrachi_Bats_2023.A3 |
| Fort Campbell Army Base | Tennessee | Austin Peay State University | 20.003R | Tennessee Wildlife Resources Agency 2314, and Kentucky Department of Fish and Wildlife Resources SC2411143 |

**Table S2.** Bat species-specific foraging home range areas associated with bat species captured in summer 2023 for this pilot project. Foraging homerange area was unknown for *Myotis occultus*, so the foraging homerange area for the closely related *Myotis lucifugus* was used.

| **Species** | **Foraging homerange distance (km)** | **Foraging distance category** | **Foraging homerange area (km^2^)** | **Reference** |
| --- | --- | --- | --- | --- |
| *Antrozous pallidus* | 5 | short (< 10 km) | 79 | [Ball 2002; Frick et al. 2009](https://www.zotero.org/google-docs/?TBnFmW) |
| *Eptesicus fuscus* | 3 | short (< 10 km) | 29 | [Menzel et al. 2001](https://www.zotero.org/google-docs/?5L63jJ) |
| *Lasiurus cinereus* | 6 | short (< 10 km) | 113 | [Barclay 1989](https://www.zotero.org/google-docs/?T12xzd) |
| *Myotis occultus** | 3.8 | short (< 10 km) | 45 | range for *Myotis lucifugus*, [Randall et al. 2014](https://www.zotero.org/google-docs/?C1UJBK) |
| *Lasiurus borealis* | 1 | short (< 10 km) | 3 | [Elmore et al. 2005; Walters et al. 2007](https://www.zotero.org/google-docs/?1KyNR8) |
| *Tadarida brasiliensis* | 40 | long (> 10 km) | 5027 | [Davis et al. 1962](https://www.zotero.org/google-docs/?qcGdPw) |
| *Nycticeius humeralis* | 1 | short (< 10 km) | 6 | [Morris et al. 2011; Hall and Bennett 2021](https://www.zotero.org/google-docs/?tzdjRd) |

*Information on foraging home range distance or area of *Myotis occultus* were not available in the literature, so we instead used information from *Myotis lucifugus*, a closely related species [(Piaggio et al. 2002)](https://www.zotero.org/google-docs/?Ngwego).

**Table S3.** Loadings for the two principal components of land use proportions within a bat species’ foraging home range area. Land use categories are from the US Geological Survey’s 2019 National Land Cover Database [(Dewitz and US Geological Survey 2021)](https://www.zotero.org/google-docs/?1Xq0mK).

| **Land Use Category** | **PC1** | **PC2** |
| --- | --- | --- |
| Developed, Open Space | -0.35 | -0.03 |
| Developed, Medium Intensity | -0.34 | -0.07 |
| Developed, Low Intensity | -0.34 | 0.06 |
| Developed, High Intensity | -0.34 | -0.08 |
| Deciduous Forest | -0.33 | -0.10 |
| Mixed Forest | -0.33 | -0.12 |
| Woody Wetlands | -0.31 | -0.09 |
| Hay/Pasture | -0.23 | -0.08 |
| Evergreen Forest | 0.01 | 0.41 |
| Shrub/Scrub | 0.01 | 0.41 |
| Cultivated Crops | 0.13 | -0.38 |
| Emergent Herbaceous Wetlands | 0.16 | -0.37 |
| Open Water | 0.16 | -0.37 |
| Herbaceous | 0.17 | -0.36 |
| Barren Land | 0.26 | 0.24 |

**Table S4.** We created 20 generalized linear models (GLMs) for neutrophil:lymphocyte ratios (NL ratios) and determined the most supported candidate models based on Akaike information criterion corrected for small sample size (AICc) and Akaike weights (*w_i_*). *k* indicates the number of estimated parameters from each model. Predictor variables are represented as follows: Hg_mg_kg = individual total Hg concentrations from bat fur (mg/kg); mn_intensity = individual red blood cell micronuclei intensity (micronuclei/150,200 red blood cells); gltA = individual *Bartonella* spp. infection status (positive, negative); rep_stat = individual female bat reproductive status upon capture (non-reproductive, pregnant lactating); PC1 = extracted values of the first axis from a principal component analysis (PCA) condensing land use proportions from species-specific foraging home ranges surrounding capture location; PC2 = extracted values of the second axis from the same PCA; forg_dist = species-specific foraging homerange distance (short [< 10 km], long [> 10 km]; see Table S1); hold_time = time difference between individual bat blood collection and capture.

| **Model ID** | **GLM predictors** (NL ratios ~ …) | ***k*** | **ΔAICc** | **R^2^_adj_** | ***w_i_*** |
| --- | --- | --- | --- | --- | --- |
| 11 | mn_intensity + repstat + hold_time | 4 | 0.00 | 0.27 | 0.520 |
| 1 | mn_intensity*repstat + hold_time | 5 | 1.68 | 0.24 | 0.224 |
| 15 | PC1 + repstat + hold_time | 4 | 3.04 | 0.19 | 0.114 |
| 9 | forg_dist*rep_stat + hold_time | 5 | 5.23 | 0.21 | 0.038 |
| 12 | mn_intensity + gltA + hold_time | 4 | 5.46 | 0.24 | 0.034 |
| 2 | mn_intensity*gltA + hold_time | 5 | 6.48 | 0.25 | 0.020 |
| 5 | PC1*rep_stat + hold_time | 5 | 7.23 | 0.17 | 0.014 |
| 17 | PC2 + rep_stat + hold_time | 4 | 7.98 | 0.06 | 0.010 |
| 19 | forg_dist + rep_stat + hold_time | 4 | 7.99 | 0.06 | 0.010 |
| 13 | Hg_mg_kg + rep_stat + hold_time | 4 | 8.83 | 0.05 | 0.006 |
| 16 | PC1 + gltA + hold_time | 4 | 9.80 | 0.12 | 0.004 |
| 6 | PC1*gltA + hold_time | 5 | 10.07 | 0.14 | 0.003 |
| 7 | PC2*rep_stat + hold_time | 5 | 10.80 | 0.07 | 0.002 |
| 3 | Hg_mg_kg*rep_stat + hold_time | 5 | 13.49 | < 0.01 | 0.001 |
| 18 | PC2 + gltA + hold_time | 4 | 14.51 | < 0.01 | 0.000 |
| 20 | forg_dist + gltA + hold_time | 4 | 15.61 | < 0.01 | 0.000 |
| 14 | Hg_mg_kg + gltA + hold_time | 4 | 15.77 | < 0.01 | 0.000 |
| 8 | PC2*gltA + hold_time | 5 | 17.05 | < 0.01 | 0.000 |
| 10 | forg_dist*gltA + hold_time | 5 | 17.09 | 0.02 | 0.000 |
| 4 | Hg_mg_kg*gltA + hold_time | 5 | 17.81 | < 0.01 | 0.000 |


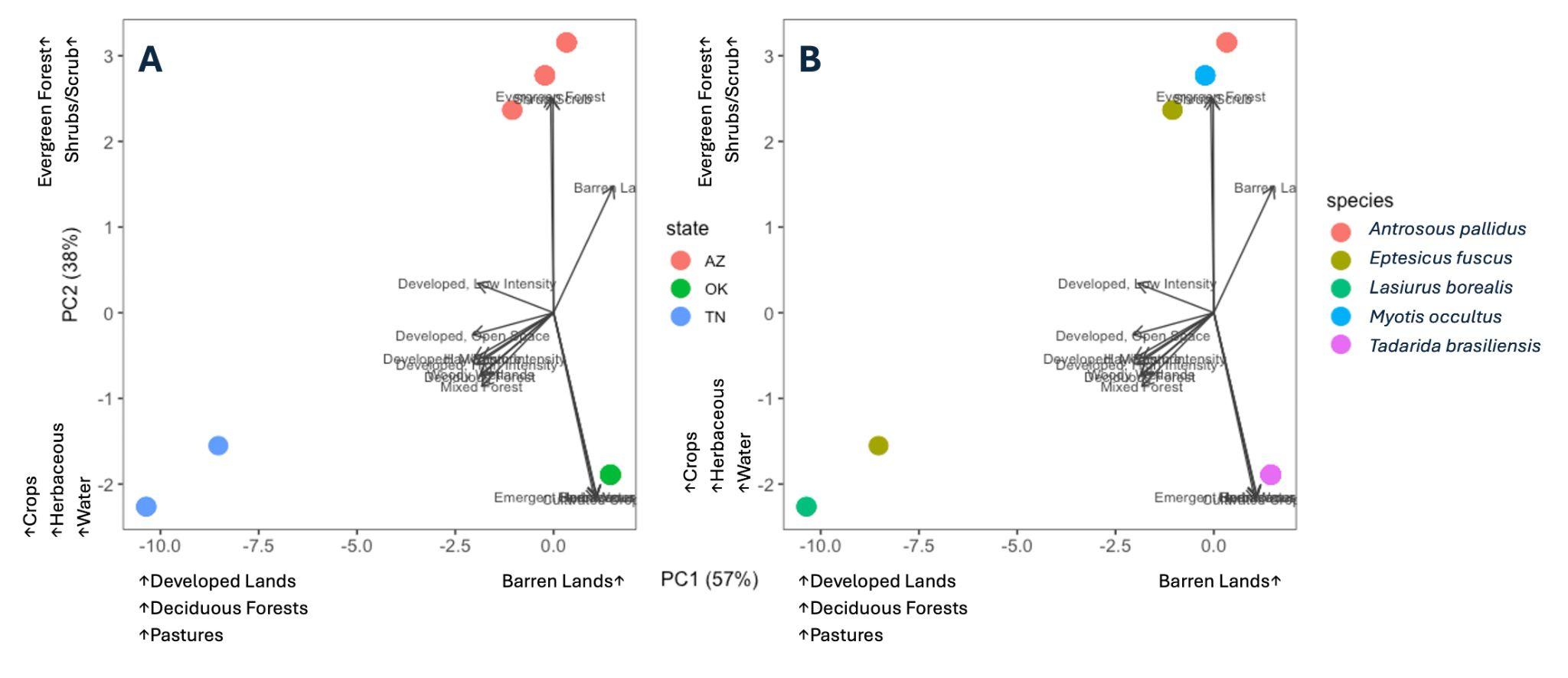


**Figure 1**. Ordinal plots for land use proportions within species-specific foraging home ranges for North American bats. Plots are grouped by **A)** state of capture or **B)** species identification. Axes (PC1 or PC2) are summarized by land use types that most heavily loaded onto each axis.

**References**

[Ball LC. 2002. A Strategy for Describing and Monitoring Bat Habitat. The Journal of Wildlife Management 66:1148–53.](https://www.zotero.org/google-docs/?v7vGTY)

[Barclay RMR. 1989. The effect of reproductive condition on the foraging behavior of female hoary bats, *Lasiurus cinereus*. Behav Ecol Sociobiol 24:31–37.](https://www.zotero.org/google-docs/?v7vGTY)

[Davis RB, Herreid CF, Short HL. 1962. Mexican Free-Tailed Bats in Texas. Ecological Monog 32:311–46.](https://www.zotero.org/google-docs/?v7vGTY)

[Dewitz J, US Geological Survey. 2021. National Land Cover Database (NLCD). Products (version 3.0, February 2024).](https://www.zotero.org/google-docs/?v7vGTY)

[Elmore LW, Miller DA, Vilella FJ. 2005. Foraging Area Size and Habitat Use by Red Bats (*Lasiurus borealis*) in an Intensively Managed Pine Landscape in Mississippi. The American Midland Naturalist 153:405–17.](https://www.zotero.org/google-docs/?v7vGTY)

[Frick WF, Heady PA III, Hayes JP. 2009. Facultative Nectar-Feeding Behavior in a Gleaning Insectivorous Bat (*Antrozous pallidus*). Journal of Mammalogy 90:1157–64.](https://www.zotero.org/google-docs/?v7vGTY)

[Hall EM, Bennett VJ. 2021. Seasonal variation in home range size of evening bats (*Nycticeius humeralis*) in an urban environment. Journal of Mammalogy 102:1497–1506.](https://www.zotero.org/google-docs/?v7vGTY)

[Menzel MA, Carter TC, Jablonowski LR, Mitchell BL, Menzel JM, Chapman BR. 2001. Home Range Size and Habitat Use of Big Brown Bats (*Eptesicus fuscus*) in a Maternity Colony Located on a Rural-Urban Interface in the Southeast. The Journal of the Elisha Mitchell Scientific Society 117:36–45.](https://www.zotero.org/google-docs/?v7vGTY)

[Morris AD, Miller DA, Conner LM. 2011. Home-Range Size of Evening Bats (*Nycticeius humeralis*) in Southwestern Georgia. Southeastern Naturalist 10:85–94.](https://www.zotero.org/google-docs/?v7vGTY)

[Piaggio AJ, Valdez EW, Bogan MA, Spicer GS. 2002. Systematics of *Myotis occultus* (Chiroptera: Vespertilionidae) Inferred from Sequences of Two Mitochondrial Genes. Journal of Mammalogy 83:386–95.](https://www.zotero.org/google-docs/?v7vGTY)

[Randall LA, Jung TS, Barclay RM. 2014. Roost-Site Selection and Movements of Little Brown Myotis (*Myotis lucifugus*) in Southwestern Yukon. Northwestern Naturalist 95:312–17.](https://www.zotero.org/google-docs/?v7vGTY)

[Walters BL, Ritzi CM, Sparks DW, Whitaker JO. 2007. Foraging Behavior of Eastern Red Bats (*Lasiurus borealis*) at an Urban-rural Interface. The American Midland Naturalist 157:365–73.](https://www.zotero.org/google-docs/?v7vGTY)
